# Neutralization of Diverse Human Cytomegalovirus Strains Conferred by Antibodies Targeting Viral gH/gL/pUL128-131 Pentameric Complex

**DOI:** 10.1128/JVI.02033-16

**Published:** 2017-03-13

**Authors:** Sha Ha, Fengsheng Li, Matthew C. Troutman, Daniel C. Freed, Aimin Tang, John W. Loughney, Dai Wang, I-Ming Wang, Josef Vlasak, David C. Nickle, Richard R. Rustandi, Melissa Hamm, Pete A. DePhillips, Ningyan Zhang, Jason S. McLellan, Hua Zhu, Stuart P. Adler, Michael A. McVoy, Zhiqiang An, Tong-Ming Fu

**Affiliations:** a Merck Research Laboratories, Merck and Co., Inc., Kenilworth, New Jersey, USA; b Texas Therapeutics Institute, the Brown Foundation Institute of Molecular Medicine, University of Texas Health Science Center at Houston, Houston, Texas, USA; c Geisel School of Medicine, Dartmouth College, Hanover, New Hampshire, USA; d Rutgers-New Jersey Medical School, Newark, New Jersey, USA; e CMV Research Foundation, Richmond, Virginia, USA; f Virginia Commonwealth University School of Medicine, Richmond, Virginia, USA; Oregon Health & Science University

**Keywords:** human cytomegalovirus, strain coverage, pentameric complex, epitope mapping, antibodies, neutralization, vaccines

## Abstract

Human cytomegalovirus (HCMV) is the leading cause of congenital viral infection, and developing a prophylactic vaccine is of high priority to public health. We recently reported a replication-defective human cytomegalovirus with restored pentameric complex glycoprotein H (gH)/gL/pUL128-131 for prevention of congenital HCMV infection. While the quantity of vaccine-induced antibody responses can be measured in a viral neutralization assay, assessing the quality of such responses, including the ability of vaccine-induced antibodies to cross-neutralize the field strains of HCMV, remains a challenge. In this study, with a panel of neutralizing antibodies from three healthy human donors with natural HCMV infection or a vaccinated animal, we mapped eight sites on the dominant virus-neutralizing antigen—the pentameric complex of glycoprotein H (gH), gL, and pUL128, pUL130, and pUL131. By evaluating the site-specific antibodies in vaccine immune sera, we demonstrated that vaccination elicited functional antiviral antibodies to multiple neutralizing sites in rhesus macaques, with quality attributes comparable to those of CMV hyperimmune globulin. Furthermore, these immune sera showed antiviral activities against a panel of genetically distinct HCMV clinical isolates. These results highlighted the importance of understanding the quality of vaccine-induced antibody responses, which includes not only the neutralizing potency in key cell types but also the ability to protect against the genetically diverse field strains.

**IMPORTANCE** HCMV is the leading cause of congenital viral infection, and development of a preventive vaccine is a high public health priority. To understand the strain coverage of vaccine-induced immune responses in comparison with natural immunity, we used a panel of broadly neutralizing antibodies to identify the immunogenic sites of a dominant viral antigen—the pentameric complex. We further demonstrated that following vaccination of a replication-defective virus with the restored pentameric complex, rhesus macaques can develop broadly neutralizing antibodies targeting multiple immunogenic sites of the pentameric complex. Such analyses of site-specific antibody responses are imperative to our assessment of the quality of vaccine-induced immunity in clinical studies.

## INTRODUCTION

Human cytomegalovirus (HCMV) is ubiquitous in the human population. While HCMV infection is, in general, asymptomatic in healthy individuals, it can cause severe diseases in immunocompromised patients, such as transplant recipients under immunosuppression. HCMV is also recognized as the leading cause of *in utero* viral infection, estimated to occur in approximately 0.64% of pregnancies in the United States (1). Congenital HCMV transmission can occur following primary infection in HCMV-seronegative mothers or nonprimary infection in HCMV-seropositive women (2). Although the majority of infected newborns have no clinical presentation of infection at birth, congenital HCMV infection can lead to neurodevelopmental sequelae in 12 to 25% of infected children, with manifestations that include sensorineural hearing loss and learning disabilities. No vaccine is yet available despite the fact that the Institute of Medicine has assigned the development of a prophylaxis against congenital HCMV to the highest category of vaccine priority since 1999 (3).

Preconceptional maternal immunity from natural HCMV infection is associated with a 69% reduction in the risk of maternal-fetal transmission (4). In addition, HCMV-seropositive women with a child in day care are protected against secondary infection from HCMV shed by their children (5). These observations indicate that natural HCMV immunity is protective against HCMV transmission in both vertical and horizontal settings; this notion has been adopted as the rationale for the design and development of live attenuated HCMV vaccines, such as the Towne vaccine (6–8). However, the immunity from naturally acquired infection may not provide complete protection against superinfection (9). Healthy seropositive women can acquire secondary infection, diagnosed either on the basis of viral shedding or by inference from serological responses to antigens different from those induced by their prior HCMV infection (10). Importantly, superinfection in women can lead to congenital transmission (11, 12), and children born with such congenital infections can develop sequelae similar to, but usually milder than, those caused by primary maternal infection (13, 14). The lack of complete protection by natural immunity may be due to defective host cellular immunity to HCMV, as documented in transplant recipients under immunosuppression. It may also be due to exposure to viral inocula of high infectivity, such as those found in the urine and saliva of toddlers (15). Lastly, antiviral antibodies induced by natural infection may have strain specificity, and under this circumstance, the preconceptional maternal immunity may not be effective to protect against the congenital transmission of a different HCMV strain. Understanding the strain coverage of antibody responses has important implications for vaccine development.

HCMV is a double-stranded DNA virus with a genome capacity to encode at least 20 glycoproteins (16, 17). Entry of HCMV requires the concerted efforts of multiple glycoprotein complexes. Glycoprotein B (gB) is a class III fusion protein (18–20). Its fusogenic activity must be triggered via interaction with complexes containing glycoproteins H (gH) and L (gL) (18, 21, 22). A trimeric complex that includes gO (gH/gL/gO) mediates viral entry into fibroblasts, and recent reports suggest that gH/gL/gO might be involved in viral entry into all cell types (23–25). The pentameric complex composed of gH/gL bound with pUL128, pUL130, and pUL131 determines viral tropism for epithelial cells, endothelial cells, and leukocytes, most likely through a receptor-mediated endocytosis pathway (20, 26–31). *In vitro* characterization of purified monoclonal antibodies (MAbs) reveals two categories of antiviral antibodies: one neutralizes infection of epithelial cells and predominantly recognizes epitopes located on the pentameric complex, whereas the other neutralizes infection of fibroblasts as well as epithelial cells and recognizes epitopes located either on gB or the gH/gL/gO complex (27, 32–35). It is unknown which category of antibodies is more important in preventing HCMV infection *in vivo*.

We recently reported a vaccine comprised of a replication-defective AD169 variant strain in which the pentameric complex was restored. The candidate, named V160, is currently under clinical evaluation and has been shown capable of eliciting both humoral and cell-mediated immune responses in preclinical animal models (36). Importantly, it is designed to present all relevant antigens, including the pentameric complex, to the immune system in their natural conformations. To address the issue of strain coverage by V160, we assembled a panel of neutralizing MAbs targeting the pentameric complex. Derived from one vaccinated rabbit and three naturally infected healthy human donors, these MAbs were used as probes for the identification of eight immunogenic sites on the pentameric complex. Antibodies with specificity to seven of these sites can neutralize a panel of HCMV primary isolates. Furthermore, immune sera from V160-vaccinated rhesus macaques were capable of competing against these neutralizing MAbs, with effective strain coverage as assessed by neutralization.

## RESULTS

### Biochemical properties of the pentamer-specific antibodies.

Previously, we identified 11 elite neutralizing MAbs from a vaccinated rabbit that recognized the pentameric complex (27). Later, we identified and cloned 10 antibodies based on neutralization from memory B cells isolated from three healthy human donors with natural HCMV infection, and they were found to be pentamer-specific MAbs. We assembled these neutralizing MAbs, including 10 human and 11 rabbit MAbs, in order to investigate unique neutralizing epitopes on this antigen complex.

**FIG 1:**
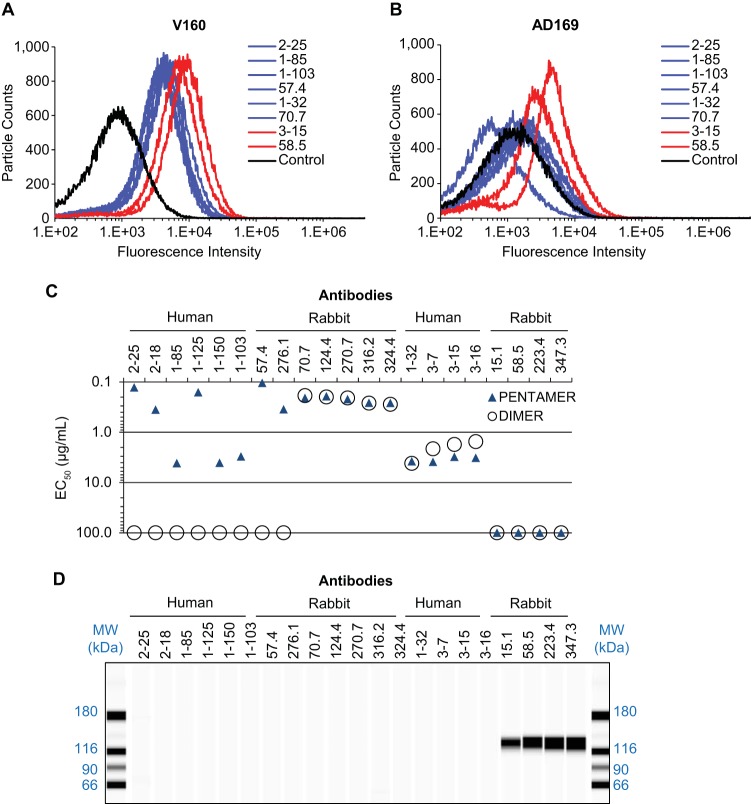
Biochemical characterizations of MAbs specific for the pentameric complex. (A and B) To evaluate the reactivity of selected MAbs to virus particles measured by flow cytometry, V160 virus with restored expression of the pentameric gH complex (A) and AD169 virus (B) was mixed with each MAb as indicated and then stained with fluorescence-labeled secondary antibody. The control samples were incubated with polyclonal antibodies from a seronegative donor. Antibodies that bound to both viruses are indicated in red. The data shown are representative of two experiments. (C) Relative binding affinity was determined by quantitative ELISA and is expressed as EC_50_, which is defined as the IgG concentration needed to achieve 50% maximal binding signal. EC_50_s were determined by four-parameter curve fitting, and if there was poor fit, an arbitrary EC_50_ of 100 μg/ml was assigned. Recombinant pentameric complex (PENTAMER) or gH/gL homodimer (DIMER) were made based on the viral sequence of the Towne strain. (D) Western blot analysis of MAbs to denatured and reduced AD169 virus antigens.

We first confirmed by flow cytometry that the selected MAbs from the panel can recognize the pentameric complex in its native form on viral particles (37). V160 virus, restored with pentameric complex expression, can be labeled by all the MAbs tested (Fig. 1A). In contrast, AD169 virus, which lacks the expression of the pentameric complex, cannot be labeled by most MAbs except 58.5 and 3-15 (Fig. 1B). This result suggested that there exist some common epitopes between the gH/gL/gO and the pentameric complex.

To better understand the antibody specificity to the common gH/gL stalk or a region unique to the pentameric complex, we tested the binding of these MAbs to the soluble forms of pentameric complex gH/gL/pUL128-131 (where pUL128-131 represents pUL128, pUL130, and pUL131) and the gH/gL dimer, which are referred to, respectively, as the pentamer and the dimer. Both recombinant complexes were previously demonstrated to retain the conformational neutralizing epitopes as antigens comparable to the membrane-bound forms (38). The relative affinities of these MAbs to the antigens were assessed and calculated as the effective concentration of IgG needed to achieve 50% of maximal signal in ELISA (EC_50_). Nine antibodies could bind to both the pentamer and the dimer (Fig. 1C), suggesting their specificity to the gH/gL portion of the complex, and eight could bind only to the pentamer, suggesting their specificity to an epitope involving at least one of the three components of pUL128-131. Four antibodies (15.1, 58.5, 223.4, and 347.3) did not react to either the pentamer or the dimer in ELISA. In addition, it is worth noting that two gH/gL binders, 1-32 and 70.7, did not label AD169 virus (Fig. 1A and B), even though these two epitopes should in theory present in the gH/gL/gO complex. Thus, the inability of antibodies 1-32 and 70.7 to react to AD169 virus suggested that their epitopes on the gH/gL/gO complex were not readily accessible on the viral envelope, possibly due to the interference of gO.

We then measured the reactivity of these MAbs to denatured viral proteins by Western blotting, with the assumption that viral proteins, after treatment with detergent and reducing agent, would be devoid of any conformational epitope. In Fig. 1D, none of 17 MAbs reacted to any viral proteins, suggesting that they likely targeted conformational epitopes. Four MAbs (15.1, 58.5, 223.4, and 347.3) reacted to an antigen of about 125 kDa from denatured V160 virus in Western blots. These MAbs were subsequently identified to react to the linear epitopes located at gH residues 26 to 43 that are specific to AD169 (data not shown). This result was consistent with the observation that these MAbs did not react to either the pentamer or the dimer, since both recombinant complexes used in ELISA were constructed with the gH based on the Towne strain amino acid sequence (27).

### Mapping the immunogenic sites by antibody binding competition.

To further group this panel of antibodies on the basis of their recognition sites, we used biolayer interferometry to measure the pairwise antibody binding competition. The competition results unveiled seven unique immunogenic sites of the pentamer (Table 1). Among the seven immunogenic sites, only site 5 partially overlapped with site 6, while no competition was observed among sites 1, 2, 3, 4, 6, and 7, suggesting that these sites contain nonoverlapping epitopes. We did not include MAb 1-125 in this experiment since our earlier experiment using individual biotinylated MAbs as probes in competition ELISA showed that MAb 1-125 competed effectively with site 2 antibodies 1-85 and 1-150 (data not shown).

**TABLE 1:**
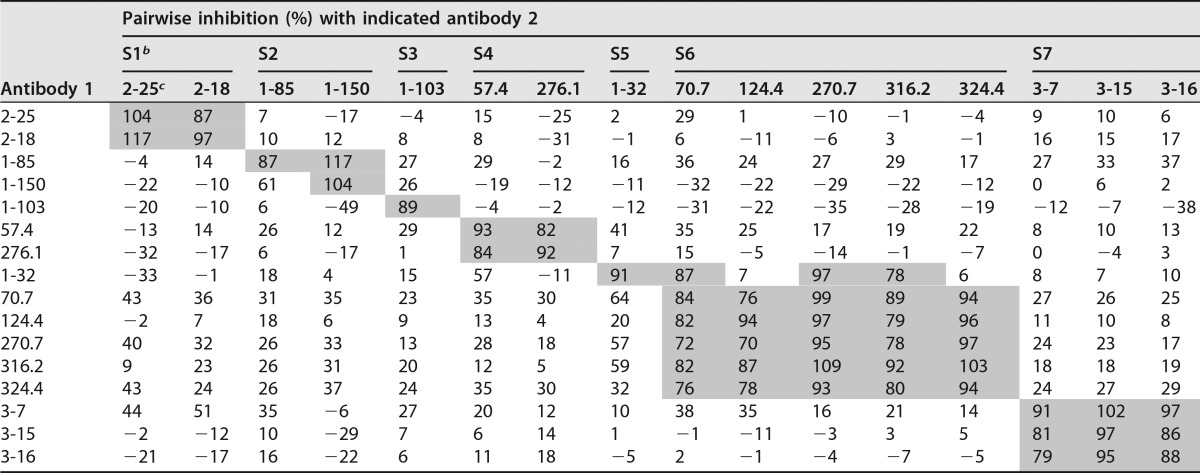
Summary of pairwise antibody inhibition^*a*^

a Biosensors coated with recombinant soluble pentameric complex were mock treated (PBS) or saturated with 15 μg/ml antibody 1 prior to exposure to 15 μg/ml antibody 2. If antibodies 1 and 2 compete for binding, binding of antibody 2 will be decreased by pretreatment with antibody 1 in comparison to mock treatment. The percentage of inhibition for each antibody 2 was calculated by normalizing these signal decreases to the total binding signal in mock treatment. Inhibition of ≥70% is shaded. Negative signal indicated that antibody 2 binding increased in the presence of antibody 1. It could be caused by the synergetic binding between two independent epitopes or irrelevant antibody-antibody interaction.

^b^ S1, site 1, etc.

^c^ Antibody.

### Epitope mapping by electron microscopy (EM).

**FIG 2:**
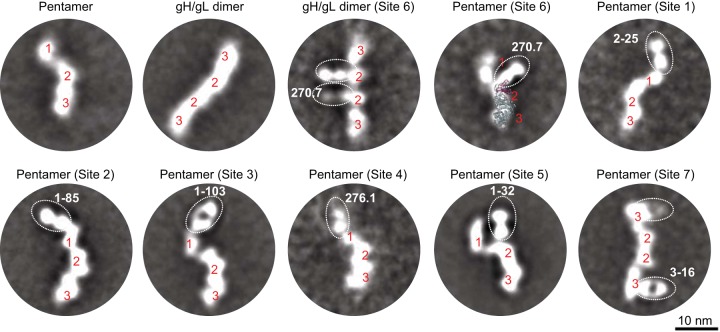
Negative-staining EM 2D class averages of recombinant pentamer and gH/gL homodimer and their complexes with various Fabs. Three domains of the pentamer are labeled with red numbers. The EBV gH/gL structure (PDB 3PHF) is shown as ribbons, with gL colored magenta and gH colored gray. The images within the dotted ovals represent the indicated Fab.

To confirm and map the multiple immunogenic sites on the pentamer revealed by biochemical characterizations, we analyzed negative-staining EM two-dimensional (2D) class averages of the pentamer bound by representative antibodies from sites 1 to 7 (Fig. 2). The 2D class averages illustrated that the free pentamer contained three distinguishable domains with a curved domain 1 loosely connected to a stalk region of domains 2 and 3, approximately 4 nm and 7 nm in length, respectively. We discovered that Fab 270.7 bound to the pentamer at domain 2, while this Fab also bound to the gH/gL homodimer at the inner domain, which was known to comprise gL and the N terminus of gH (33). It suggested that domain 1 was likely comprised of pUL128-131, domain 2 of gL and the N terminus of gH, and domain 3 of the C terminus of gH, consistent with a previous report (33). The crystal structure of Epstein-Barr virus (EBV) gH/gL (PDB 3PHF), which shares 24% sequence similarity with HCMV gH/gL (39), can be overlaid on the 2D class image of pentamer-270.7, supporting the assignment for domains 2 and 3. Site 1 to 4 antibodies were found to target the tip of domain 1, consistent with the observation that site 1 to 4 antibodies bound to the pentamer only and not to the gH/gL stalk region (Fig. 1C). Site 5 MAb 1-32 targeted one end of domain 2, while site 6 MAb 270.7 targeted the other side of domain 2. Site 7 MAb targeted the side of domain 3. Since domains 2 and 3 were preserved in gH/gL, the 2D images were consistent with the observation that site 5 to 7 antibodies bound to both the pentamer and the gH/gL homodimer (Fig. 1C).

**FIG 3:**
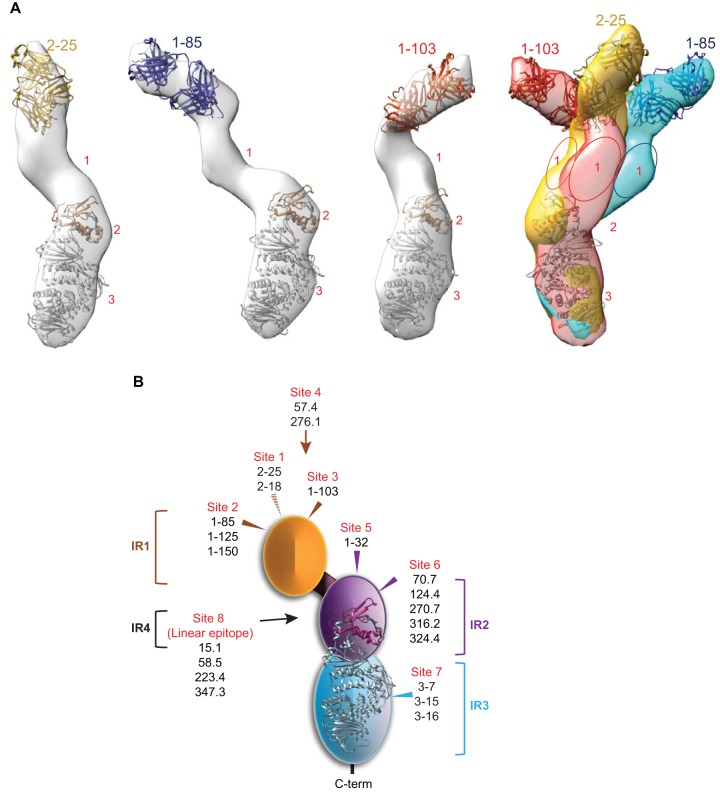
EM 3D reconstruction of pentamer bound by Fabs and a summary diagram of identified immunogenic sites. (A) Structures of the pentamer bound with Fab 2-15, Fab 1-85, or Fab 1-103 and their overlay, which was rotated 180° horizontally with respect to the individual structures. The random conical tilt (RCT) method was used to reconstruct the 3D structures. The surface rendering was generated using the Chimera visualization package. To aid the interpretation of the structure, the crystal structures of EBV gH/gL (PDB 3PHF) and anti-gB Fab (PDB 4OSU) were manually fitted into the EM 3D map by use of Chimera. Glycoprotein gH is colored gray, and gL is colored yellow. The numbers 1 to 3 correlate with the domains visible in the EM 2D class averages (Fig. 2). (B) A diagram showing the four immunogenic regions (IRs) and eight immunogenic sites of the HCMV pentamer targeted by 20 neutralizing antibodies. The arrows show the approximate positions of the immunogenic sites based on EM images.

Three-dimensional (3D) reconstruction analyses were performed for the pentamer bound by Fab 2-25, 1-85, and 1-103, and the 3D reconstructed density maps at a resolution of 35 to 40 Å are shown in Fig. 3A. The crystal structures of EBV gH/gL (PDB 3PHF) and anti-gB Fab (PDB 4OSU) were manually fitted into the EM 3D structure to facilitate the interpretation (39, 40). By overlaying the gH/gL stalks of the three images (Fig. 3A, left three diagrams), we found that only domain 1 could adopt different orientations in these complexes, suggesting its orientation flexibility (Fig. 3A, right panel), consistent with a previous report (34). By comparing the three structures with those reported previously (EMD-6436, -6347, -6438), we discovered that site 3 is a unique site that has not been described before. Sites 1 and 2 are close to the previously reported binding site recognized by 2C12 (34), and a competitive binding study will be needed for a definitive comparison.

From the biochemical characterizations, pairwise antibody competition, and EM epitope mapping, a total of four immunogenic regions (IR) on the pentameric complex could be assigned (Fig. 3B). IR1 was composed of pUL128, pUL130, and pUL131, shown as domain 1 in the pentamer EM 2D averages. Four nonoverlapping conformational immunogenic sites (sites 1 to 4) were identified in IR1 from our MAb collection, and an additional 3 or 4 unique sites exist in IR1 from other reports (34). IR2 was composed of gL and the N terminus of gH, shown as domain 2 in the pentamer EM 2D averages. Two partially overlapping conformational immunogenic sites were identified in IR2 (sites 5 and 6). IR3, shown as domain 3 in the pentamer 2D averages, consisted of the C terminus of gH with one immunogenic site (site 7). IR4 resided in the first 40 amino acids of gH with one linear immunogenic site (site 8), commonly used to distinguish between AD169 and Towne strains (41).

### Differential inhibition of viral entry into ARPE-19 and MRC-5 cells.

**FIG 4:**
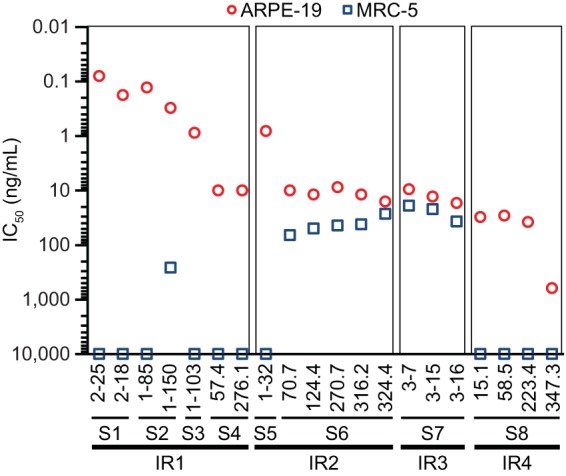
Potencies of IR1 to IR4 antibodies in neutralizing HCMV in ARPE-19 and MRC-5 cells. Representative antibodies to each immunogenic site (and IR) were incubated in titration with HCMV. The mixtures were then applied to ARPE-19 cells (red circles) or MRC-5 cells (blue squares), viral entry events were documented by determining viral immediate early gene expression, and IC_50_s, defined as the IgG concentration to achieve 50% viral entry inhibition, were calculated using four-parameter curve fitting. The data shown are representative of three experiments.

We next evaluated the neutralizing function of antibodies toward each identified site, with the speculation that each site might be engaged differently in the viral entry process. We tested the neutralization potencies of these antibodies in both ARPE-19 and MRC-5 cells (Fig. 4).

As expected, IR1 antibodies, all specific to the pentamer, demonstrated the most potent antiviral function in ARPE-19 cells, with a potency more than approximately a thousandfold higher than that of CMV hyperimmune globulin (CMV-HIG). However, this class of antibodies could not inhibit viral entry in MRC-5 cells as previously reported for the potent neutralizing antibodies against viral epithelial entry (27).

IR2 and IR3 antibodies, with the exception of 1-32, inhibited viral entry in both ARPE-19 and MRC-5 cells, although they were approximately 100-fold less potent than the majority of IR1 antibodies in ARPE-19 cells, consistent with previous observations (27, 32, 33, 35). The fact that IR2 and IR3 antibodies showed similar potencies in both APRE-19 and MRC-5 cells suggested that IR2 and IR3 might be involved in a common viral entry mechanism, such as interacting with gB, independent of cell tropism. 1-32 was a unique IR2 MAb with no antiviral activity in fibroblasts. The EM 2D class average analysis showed that Fab 1-32 was directed at domain 2 at an approximate 180° angle against the gH/gL stalk, a binding mode significantly different from that of other gH/gL antibodies, such as 124.4 and 270.7 (Fig. 2). We speculated that although its binding site was within the gH/gL domain, the unique binding angle may indicate that the epitope of 1-32 is outside the region involved in viral entry in fibroblasts, such as the interaction with gB.

IR4 antibodies in this study inhibited viral entry into APRE-19 but not MRC-5 cells. These antibodies were apparently different from the AP86 binding sera that bind to the gH N-terminal linear epitope(s) and neutralize AD169 in fibroblasts (42). This may suggest that there are multiple epitopes within the gH N-terminal region, including those recognized by IR4 antibodies, and the neutralization mechanism for these antibodies may be complex and cell type specific.

The neutralization potencies of these antibodies in different cell types suggested that IR1, the domain essential for viral entry in epithelial cells, was responsible for the highly potent antibody responses and that IR2 and IR3 were necessary for antibody responses to protect different cell types, including fibroblasts. All three IRs are critical regions to be included in rational vaccine design.

### Conservation of immunogenic sites among different HCMV strains.

To address the question about antibody-mediated coverage of HCMV strains, we next determined whether the IR-specific antibodies could neutralize genetically defined clinical isolates. Eleven isolates with full-length genome information were selected and cultured in ARPE-19 cells (Table 2). For example, these isolates were diverse in gO sequences, as previously reported (43), and the average amino acid distance for gO among these strains to that in V160 was calculated to be 20.3% (Table 3). However, these strains shared relatively high similarity to the vaccine strain when their sequences of the pentameric complex were compared, with amino acid distances averaging 0.2 to 2.3% (Table 3).

**TABLE 2:** Clinical isolates and laboratory HCMV strains

Strains	GenBank accession no.	Source of virus or reference(s)
VHL/E	KX544841	55, 56
VR1814	GU179289	28, 57, 58
VR3908	KX544833	9
VR7863	KX544838	9
VR5235	KX544837	9
VR5022	KX544835	9
UxcA	KX544840	59
NR	KX544831	Isolated from a kidney transplant recipient and cloned in BAC
TB40/E	EF999921	60, 61
SUB 22	KX544834	Isolated from a urine sample of a congenitally infected neonate
SUB 24	KX544832	Isolated from a urine sample of a congenitally infected neonate
beMAD		AD169 strain from the UK (62–64) and BAC cloned and repaired for epithelial tropism (36)
TS15-rR		59

**TABLE 3:** Protein distance analysis on selected antigens between clinical isolates and V160

Clinical isolate	% Similarity with indicated V160 antigen^*a*^						
	gB	gO	gH	gL	pUL128	pUL130	pUL131
VHL/E	4.1827	23.2435	3.6864	1.9343	0.6098	1.9075	0.0002
VR1814	5.1331	15.4296	0.6727	1.9313	1.2091	2.4834	0.0486
VR3908	4.1849	20.2599	3.2649	1.9343	1.2091	2.8977	0.0074
VR7863	4.1849	22.9828	3.6831	1.5545	1.8242	1.6592	0.0071
VR5235	4.1849	20.5298	2.5922	1.5545	1.2093	2.8766	0.0069
VR5022	4.2992	22.9828	3.6831	1.5545	1.8242	0	0.0053
UXCA	4.0721	20.3977	0.5386	0.7754	1.8242	0.78	0.0045
NR	4.5172	28.365	3.4305	1.9343	1.2091	3.3916	0.7784
TB40E	4.4039	15.4163	0.535	1.5545	1.8242	1.6592	0.0038
SUB 22	4.2992	22.9828	3.6831	1.9343	1.8242	0	0
SUB 24	4.2992	22.9828	3.6831	2.3307	1.8242	0	0
Global	3.520 ± 0.007	20.255 ± 0.029	2.259 ± 0.006	1.582 ± 0.003	1.362 ± 0.003	1.340 ± 0.005	0.205 ± 0.001

a Protein distances were estimated using the algorithm within PhyML using a PAM model of molecular evolution. The values in the table were converted from a distance by taking 1 minus the distance and multiplying it by 100 to arrive at a percent similarity. Global values are the average percentage of similarity among complete viral sequences obtained from NCBI GenBank (*n* = 194).

**FIG 5:**
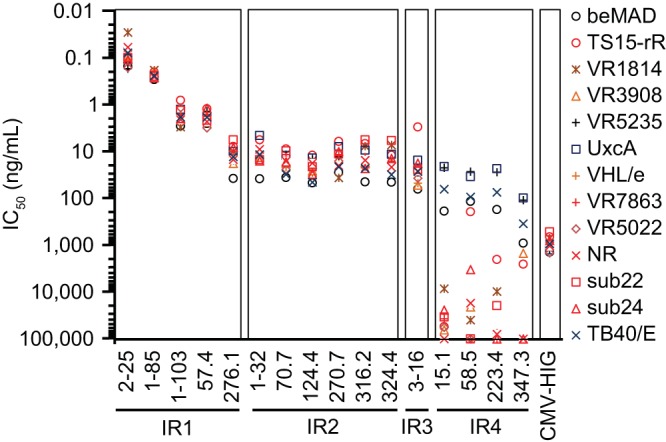
Potencies of antibodies in neutralizing 11 clinical isolates and 2 laboratory strains in ARPE-19 cells. Representative antibodies from each immunogenic site were incubated in titration with HCMV virus and then applied to ARPE-19 cells. The viral entry events were documented by immune staining of viral immediate early gene expression 24 h later. IC_50_s shown on the *y* axis were calculated using four-parameter curve fitting. Antibody designations and their classification to IR regions are marked on the *x* axis. CMV-HIG was included as a reference. Strain information is given in Table 2. The data shown are representative of two experiments.

These isolates, along with two laboratory strains, were then tested in viral neutralization assays in ARPE-19 cells (Fig. 5). IR1, IR2, and IR3 antibodies neutralized all HCMV strains tested, consistent with the sequence similarity analysis of the pentameric complex and suggesting that sites 1 to 7 were highly conserved among these strains. In contrast, IR4 antibodies showed variable potencies against different strains, suggesting that antibodies to site 8 were strain specific. Because of its strain specificity, synthetic peptides corresponding to site 8 have been used as tools for serological confirmation of superinfection (41). The neutralization of these viral strains was consistent with the observation that most of our antibodies were screened and selected based on neutralization and were found to target the antigens constituting the pentameric complex, with none to the highly variable antigens such as gO. Lastly, site 6 and 7 antibodies were effective against these strains, suggesting protection against infection of these strains in MRC-5 cells. These results confirmed the importance of sites 1 to 7 in the design of HCMV vaccines for broad strain coverage.

### Site-specific antibody responses in nonhuman primates following V160 vaccination.

**FIG 6:**
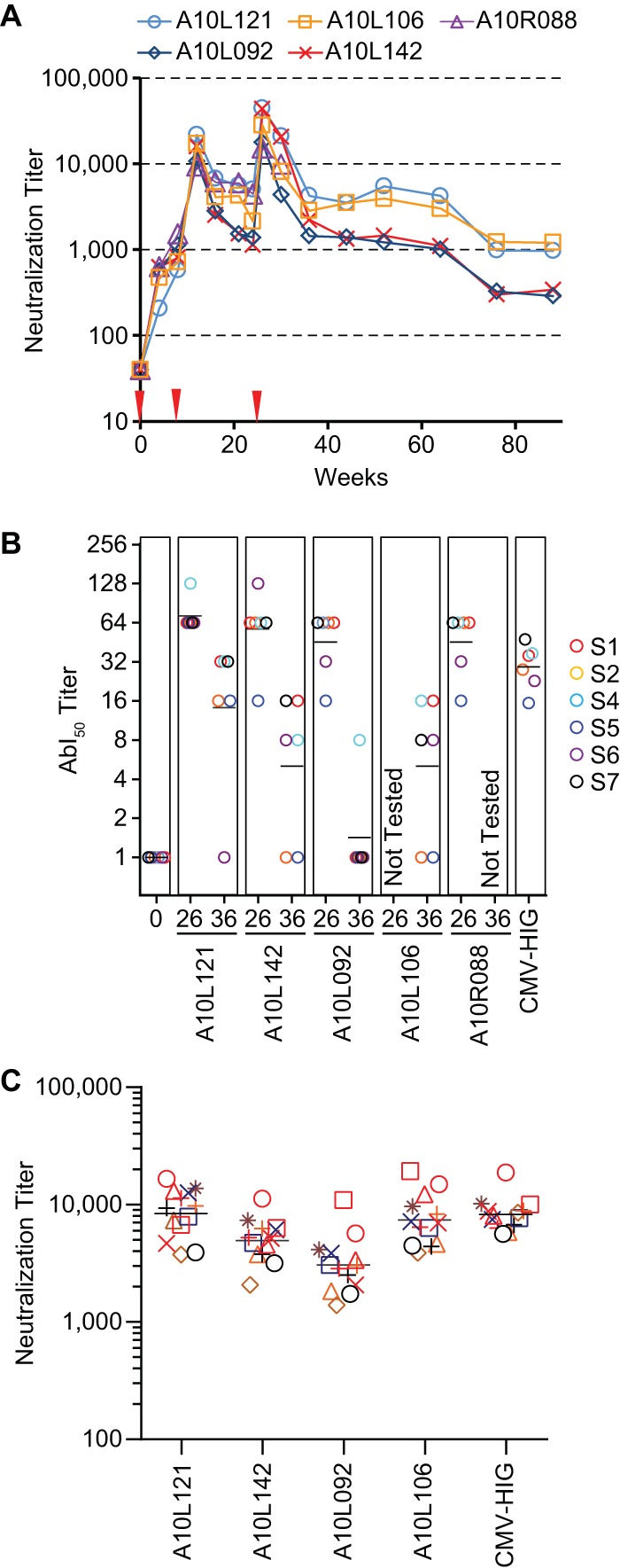
Evaluation of V160-induced antibody responses in rhesus macaques. (A) Neutralization titers of five rhesus macaques immunized with V160 HCMV vaccine. The vaccine was administered at week 0, 8, and 24 (red arrowheads), and sera collected at the indicated time points were evaluated for neutralizing activities in ARPE-19 cells. The neutralizing titers were determined by reciprocal serum dilutions to achieve 50% viral entry reduction. The data shown are representative of two experiments. (B) AbI_50_ titers of rhesus immune sera in comparison with CMV-HIG. An arbitrary number of 1 was assigned to the serum if there was no detectable activity. The horizontal black bars represent the geometric mean AbI_50_ titers to six sites. Sera from A10L106 at week 26 and A10R088 at week 36 were not available for testing. (C) Neutralization titers of four immune sera at week 36 against 11 clinical isolates and 2 laboratory strains in ARPE-19 cells. The legend symbols for HCMV isolates are as shown in Fig. 5. In panels B and C, CMV-HIG values are normalized to a starting concentration of 10 mg/ml to approximate the IgG concentration in serum.

The functional map of the seven conserved immunogenic sites enabled us to assess the quality of vaccine-induced antibody responses. As an example, five rhesus macaques were immunized at weeks 0, 8, and 24 with the replication-defective V160 vaccine formulated with Iscomatrix adjuvant. Immune sera were evaluated in viral neutralization assays in ARPE-19 cells. The epithelial neutralizing titers peaked after three vaccinations at week 26 and were sustained through week 64 (Fig. 6A).

To assess whether the immune sera contained antibodies with specificity to each immunogenic site, we measured the antibody-binding inhibition titer (last dilution of serum that inhibits ≥50% of the antibody binding [AbI_50_]) of each of the immune sera at weeks 0, 26, and 36 against the panel of probe antibodies from six sites. Site 3 was excluded from this analysis, as neither the rhesus immune sera nor CMV-HIG could compete effectively against MAb 1-103 binding to the pentamer in ELISA (data not shown). At week 0, none of the five monkeys had any preexisting AbI_50_ titer, whereas at week 26, all four monkeys tested demonstrated robust antibody responses to the six sites (Fig. 6B). Although at week 36, the vaccine induced-antibody responses waned in all cases, the AbI_50_ titer for site 4 was retained in four monkeys tested and the AbI_50_ titers for sites 1 and 7 were retained in three of the four monkeys tested. The results demonstrated that the vaccination elicited antibodies targeting multiple sites of the pentameric complex, suggesting the potential to protect against viral infection in epithelial cells (site 1, 2, and 4 antibodies) and fibroblasts (site 6 and 7 antibodies) based on the functional analysis of MAbs in Fig. 4.

Next, we compared the site-specific antibody responses in the rhesus immune sera with those from the natural infection in humans. Since CMV-HIG is prepared from the plasma of donors with high antibody titers against HCMV and has been evaluated for prophylaxis against congenital HCMV infection (44, 45), we measured the 50% inhibitory concentrations (IC_50_s) of CMV-HIG and calculated the AbI_50_ titers that represent the average serum titers from the natural infection (Fig. 6B). The comparison demonstrated that the antibody responses induced by V160 targeted sites similar to those from natural infection, and the peak AbI_50_ titers at week 26 were in the range of the CMV-HIG concentrations.

Not only did the AbI_50_ titer show the quality of antibody responses, it was also a good indicator of serum antiviral functions, as the geometric mean (GM) of AbI_50_ titers toward the six sites closely correlated with the neutralization titers, with a correlation coefficient of 0.93 (*R*^2^ = 0.87, *P* = 0.0008).

Lastly, we evaluated the ability of rhesus immune sera to cross-neutralize the clinical isolates. As shown in Fig. 6C, all 4 of the week 36 immune sera tested could neutralize these viral isolates, with potencies ranked similar to their AbI_50_ titers and matched closely the CMV-HIG concentrations. This result confirmed that V160 vaccination in nonhuman primates can elicit antibodies with antiviral activity against the clinical isolates and also suggested that the competition against all six probe antibodies could serve as a surrogate for assessing the ability of immune sera to neutralize potentially genetically diverse field strains.

## DISCUSSION

Because of its importance to HCMV vaccine development, we set out to analyze the quality of antiviral antibodies by vaccination compared to those by natural infection. Our work led to a map of immunogenic sites of the HCMV pentamer, a viral antigen complex known to be targeted by potent neutralizing antibodies. The abundance of immunogenic sites in IR1 was consistent with an observation reported recently (33), as well as our previous report that the soluble pentamer, and not the gH/gL homodimer, can deplete over 75% of the epithelial neutralizing activity in CMV-HIG (38).

The geometric mean AbI_50_ titer for the six sites served as an indicator of the quality of vaccine-induced antibodies, as it suggested not only antiviral potency but also ARPE-19 or MRC-5 cell type-specific protection *in vitro*. The absolute value of AbI_50_ may also be used to infer the quantity of each type of antibody in the immune sera. For example, when CMV-HIG competes with the six probe antibodies at 0.05 μg/ml, the AbI_50_ titers of CMV-HIG range from 15 to 47 and the IC_50_s of CMV-HIG in the viral neutralization assay range from 210 to 650 μg/ml. By this estimate, only a tiny fraction of total CMV-HIG (estimate, 0.01 to 0.02%) binds to each immunogenic site with a strength similar to that of the probe antibodies. Nevertheless, the marginal presence of 0.01 to 0.02% of a highly potent antibody, such as site 1 antibody, which has an IC_50_ of 0.2 ng/ml in the viral neutralization assay, may contribute to the majority of neutralization activity of CMV-HIG (IC_50_, approximately 1,000 ng/ml). Therefore, although the AbI_50_ titers appeared low in this study, they revealed infrequent but highly potent neutralizing antibodies in the immune sera.

Our collection of antibodies was derived from both humans with natural HCMV infection and an animal vaccinated with a whole virion vaccine, and their *in vitro* potencies against viral infection were benchmarked to the potency of CMV-HIG. In this regard, these antibodies are useful tools to identify the protective component within the vaccine-induced immune sera. Previously, Lilleri et al. reported that in pregnant women with primary HCMV infection, early emergence (<30 days) of an antibody response to epitopes in IR1 is associated with a significantly reduced risk of intrauterine HCMV transmission (46). Their study provided evidence that antibodies with IR1 specificity are a correlate to protection against maternal-fetal transmission. CMV-HIG has been evaluated for the prevention of congenital HCMV infection but with efficacies of 31% and 60% in two separate studies (44, 45), respectively, and our results suggest that CMV-HIG contains less than 0.02% of IR1 antibodies. In this regard, IR1-specific MAbs, if used as therapeutics, may provide better clinical outcomes than CMV-HIG.

Antibodies mapped to seven out of eight immunogenic sites of the pentamer demonstrated neutralizing coverage to a panel of 11 clinical isolates. This result suggested that antibodies from natural infection were likely protective *in vitro* against the strains causing HCMV superinfection. Thus, HCMV-seropositive individuals who succumb to superinfection may have a deficiency in their natural immunity to HCMV. A serological survey of 360 HCMV-seropositive women revealed a geometric mean neutralizing titer for the cohort of 1:7,500; however, about 5% of the subjects had neutralizing titers below 1:1,000 (47). In addition, it is not clear whether defective cell-mediated immunity in HCMV-seropositive subjects plays a role in superinfection. Second, superinfection may be related to viral titers of the inoculum. A human challenge study in which the HCMV-seropositive subjects were challenged with a pathogenic Toledo strain was conducted, and the results support this hypothesis, as HCMV seropositivity was protective against challenge inocula of 10 and 100 PFU, but not 1,000 PFU, of Toledo virus (48). Lastly, it is also possible that serum neutralizing potency measured *in vitro* may not truly reflect antiviral immunity in the host.

In conclusion, we identified four IRs containing eight immunogenic sites on the pentamer, an antigen for neutralizing antibodies. The functional characterization of these sites enabled the evaluation of the quality of vaccine-induced antibody responses. Rhesus macaques vaccinated with V160 generated diverse antibody responses to the pentamer, and their immune sera demonstrated neutralization against a panel of clinical HCMV isolates. The analysis of site-specific antibody responses presents a useful tool to analyze V160-induced immune responses for their role in vaccine efficacy against congenital HCMV infection.

## MATERIALS AND METHODS

### Antibody generation.

Rabbit MAbs were isolated from an animal immunized with AD169 revertant virus as previously reported (27). Human MAbs were isolated from memory B cells from three healthy individuals with natural HCMV infection. Briefly, memory B cells were enriched using an EasySep memory B-Cell kit (StemCell). Enriched memory B cells were cultured in limiting dilution in 96-well U-bottom plates with gamma-irradiated feeder cells expressing human CD40L in complete RPMI medium supplemented with interleukin-21 (50 ng/ml) (Invitrogen). The supernatant was collected at day 14 and screened for viral neutralization and/or binding activity as described previously (27). Total RNA from the cells in the positive wells was isolated and converted to cDNA using a reverse transcription kit (Invitrogen), and the IgG genes were recovered by PCR using primers that have been described previously (49). Recombinant antibodies were expressed by transient transfection in HEK293 cells and purified by protein A affinity chromatography (50). The purity and integrity of the antibodies were assessed by SDS-PAGE.

### Biochemical characterizations.

For Western blot analysis, denatured and reduced V160 samples were analyzed on a Simon capillary Western blot system (ProteinSimple) as previously described (51). Briefly, the V160 sample was mixed with sample buffer containing SDS and dithiothreitol (DTT) and then heated for 10 min at 70°C. The SDS-PAGE separation occurred in capillary and viral antigens that were probed with each primary antibody for 90 min and then for 60 min with secondary antibody, either anti-rabbit IgG with horseradish peroxidase (HRP) from ProteinSimple or anti-human IgG with HRP from Jackson ImmunoResearch. The chemiluminescence signal was measured at six different exposure times. For ELISA, recombinant dimer or pentamer was immobilized at 1 μg/ml in phosphate-buffered saline (PBS) on 96-well FluoroNunc MaxiSorp plates at 4°C overnight. The plates were then blocked with 3% nonfat milk in PBS–0.05% Tween 20. MAbs in titration in PBS were incubated for 1.5 h, and the plates were washed afterwards and then incubated with HRP-conjugated goat anti-rabbit or anti-human IgG (Southern Biotech) for 30 to 60 min. A fluorogenic HRP substrate, 10-acetyl-3,7-dihroxyphenoxazine (ADHP) (Virolabs), was added at 100 μl per well for 3 to 5 min to generate resorufin, and the fluorescent signals with excitation at 531 nm and emission at 595 nm were measured (Victor III; PerkinElmer). The effective concentration of MAb to achieve 50% maximal binding (EC_50_) was calculated using four-parameter curve fitting as described previously (27). Antibody binding to HCMV particles measured by flow cytometry was conducted as described previously (37). Briefly, V160 or AD169 virus preparations were incubated with each MAb, followed by the removal of the unbound MAb, incubation with Alexa Fluor 488-labeled secondary antibody, and removal of the unbound secondary antibody. The flow cytometer was triggered on the side light scatter signal from the HCMV particles. Negative human and rabbit polyclonal IgG were used as controls.

### Pairwise antibody competition using biolayer interferometry.

The competition assay was performed on an Octet HTX using NTA Biosensors (FortéBio). Antibodies were diluted to 15 μg/ml in PBS and placed into 384 tilted-bottom microplates. All biosensors were rehydrated with PBS for at least 10 min, loaded with recombinant pentamer (38) at 5 μg/ml in PBS for 900 s, and then washed in PBS for 60 s. A group of 16 biosensors was loaded for 2,000 s with either PBS as the control or antibody 1 at 15 μg/ml to achieve saturation. The biosensors were then washed in PBS for 60 s and transferred to wells containing different antibodies (antibody 2) to allow 1,500 s of total binding time. The decrease of antibody 2 association in the presence of antibody 1 was normalized by the total binding in the absence of antibody 1 (PBS control) in order to calculate the percentage of competition. The procedure was repeated for the remaining 15 antibodies as for antibody 1.

### Electron microscopy.

Fabs were generated from the selected antibodies and mixed with the pentamer or the gH/gL dimer at a 5:1 molar ratio for 1 h. The complexes were then separated by size exclusion chromatography and applied to glow discharg holey carbon grids and stained with 2% uranyl formate. The grids were imaged using an FEI Tecnai T12 electron microscope operating at 120 keV and equipped with an FEI Eagle 4k × 4k charge-coupled-device (CCD) camera. Tilt pair images (0°, 60°) were recorded using Leginon at a nominal magnification of ×67,000 with a nominal underfocus of −2 μm to −1 μm and electron doses of ∼25 to 30 e/A^2^.

Image processing and model reconstructions were performed using the Appion software package. Individual particles were selected using automated picking protocols on both untilted and tilted images, and the untilted particles were subjected to several rounds of reference-free alignment and classification using the XMIPP processing package. Random conical tilt (RCT) reconstructions were performed using particle pairs from exemplar class averages to obtain 3D maps of the complexes. The nominal resolution of the 3D maps is ∼35 to 40 Å, with the resolution criterion Fourier shell correlation equal to 0.5 (FSC_0.5_). The Chimera visualization package was used to produce the surface rendering of each complex and to fit the X-ray structure of EBV gH/gL (PDB 3PHF) into the EM maps.

### Virus stains and viral neutralization in ARPE-19 and MRC-5 cells.

AD169 revertant virus has been described previously (36, 52), and primary clinical isolates were recently isolated and cultured or obtained from James Waldman, Maria Revello, and Eain Murphy. The virus was culture adapted in ARPE-19 cells and purified by ultracentrifugation as previously described (47). The viral infectivity was assessed by a 50% tissue culture infective dose (TCID_50_) assay. The viral neutralization assay based on immunostaining was described previously (53).

### Vaccination study.

Rhesus macaques (Macaca mulatta) were maintained at the New Iberia Research Center (NIRC), New Iberia, LA. All animal studies were conducted in accordance with the *Guide for the Care and Use of Laboratory Animals*, and the study protocols were approved by Institutional Animal Care and Use committees. Rhesus macaques were anesthetized, and the vaccines were delivered intramuscularly in 0.5-ml volumes into deltoid muscles. V160 vaccine has been described previously (36) and was formulated prior to injection with 30 μg/dose Iscomatrix adjuvant provided by CSL Ltd. (Victoria, Australia).

### AbI_50_ titer.

Competition ELISA was used to determine the serum antibody-binding inhibition (AbI_50_) titer against a panel of seven antibodies (2-18, 1-125, 1-103, 57.4, 1-32, 124.4, and 3-16), each representing one immunogenic site. The recombinant pentamer complex was immobilized at 0.3 μg/ml in PBS on 96-well FluoroNunc MaxiSorp plates at 4°C overnight. The plates were then blocked with 3% nonfat milk in PBS–0.05% Tween 20. Rhesus serum in titration in PBS was mixed with 0.05 μg/ml of either biotinylated human antibody or unbiotinylated rabbit antibody, and then the mixture was incubated in the precoated plates for 1.5 h. The plates were washed afterwards and then incubated with HRP-conjugated detection agents, either streptavidin (BD Pharmingen) or goat anti-rabbit IgG (Southern Biotech), for 30 to 60 min. ADHP was added at 100 μl per well for 3 to 5 min to generate resorufin, and the fluorescent signals with excitation at 531 nm and emission at 595 nm were measured (Victor III; PerkinElmer). AbI_50_ titers were defined as the last dilution of serum that inhibits ≥50% of the antibody binding.

A similar competition ELISA was used to determine the IC_50_ of CMV-HIG in inhibiting the same panel of probe antibodies binding to their corresponding epitopes. Inhibition curves were constructed for each immunogenic site, and the four-parameter logistic curve fitting was done to extract IC_50_ (μg/ml) values. The average human serum IgG concentration of 10,000 μg/ml was then divided by the IC_50_s to derive AbI_50_ titers of CMV-HIG.

### Accession number(s).

The sequence information for some viral strains listed in Table 2 was determined by next-generation sequencing, and the sequences have been described elsewhere (54). These strains include VHL/E (GenBank accession number KX544841), VR3908 (KX544833), VR7863 (KX544838), VR5235 (KX544837), VR5022 (KX544835), UxcA (KX544840), NR (KX544831), sub 22 (KX544834), and sub 24 (KX544832).
